# Polystyrene nanoplastic and engine oil synergistically intensify toxicity in Nile tilapia, *Oreochromis niloticus*: Polystyrene nanoplastic and engine oil toxicity in Nile tilapia

**DOI:** 10.1186/s12917-024-03987-z

**Published:** 2024-04-16

**Authors:** Alaa El-Din H. Sayed, Walaa F. A. Emeish, Karima A. Bakry, Zeinab Al-Amgad, Jae-Seong Lee, Salwa Mansour

**Affiliations:** 1 Department of Zoology, Assiut University, Assiut, 71516 Egypt; 2 Department of Biotechnology, Molecular Biology Research & Studies Institute, Assiut University, Assiut, 71516 Egypt; 3 Fish Diseases Department, South Valley University, Qena, Egypt; 4 General Authority for Veterinary Services, Qena Veterinary Directorate, Qena, Egypt; 5 Department of Biological Sciences, College of Science, Sungkyunkwan University, Suwon, 16419 South Korea; 6 Zoology Department, South Valley University, Qena, Egypt

**Keywords:** Polystyrene nanoplastic, Engine oil, Nile tilapia, Fish toxicity, Hematobiochemical parameters, Cytokines, Oxidative stress

## Abstract

Polystyrene nanoplastic (PS-NPs) and Engine oil (EO) pose multiple ecotoxic effects with increasing threat to fish ecosystems. The current study investigated the toxicity of 15 days exposure to PS-NPs and / or EO to explore their combined synergistic effects on Nile tilapia, *Oreochromis niloticus* (*O. niloticus*). Hematobiochemical parameters, proinflammatory cytokines, and oxidative stress biomarkers as well as histological alterations were evaluated. The experimental design contained 120 acclimated Nile tilapia distributed into four groups, control, PS-NPs (5 mg/L), EO (1%) and their combination (PS-NPs + EO). After 15-days of exposure, blood and tissue samples were collected from all fish experimental groups. Results indicated that Nile tilapia exposed to PS-NPs and / or EO revealed a significant decrease in almost all the measured hematological parameters in comparison to the control, whereas WBCs and lymphocyte counts were significantly increased in the combined group only. Results clarified that the combined PS-NPs + EO group showed the maximum decrease in RBCs, Hb, MCH and MCHC, and showed the maximum significant rise in interleukin-1β (IL-1β), and interleukin-6 (IL-6) in comparison to all other exposed groups. Meanwhile, total antioxidant capacity (TAC) showed a significant (*p* < 0.05) decline only in the combination group, whereas reduced glutathione (GSH) showed a significant decline in all exposed groups in comparison to the control. Both malondialdehyde (MDA) and aspartate aminotransferase (AST) showed a significant elevation only in the combination group. Uric acid showed the maximum elevation in the combination group than all other groups, whereas creatinine showed significant elevation in the EO and combination group when compared to the control. Furthermore, the present experiment proved that exposure to these toxicants either individually or in combination is accompanied by pronounced histomorpholgical damage characterized by severe necrosis and hemorrhage of the vital organs of Nile tilapia, additionally extensively inflammatory conditions with leucocytes infiltration. We concluded that combination exposure to both PS-NPs and EO caused severe anemia, extreme inflammatory response, oxidative stress, and lipid peroxidation effects, thus they can synergize with each other to intensify toxicity in fish.

## Introduction

Nile tilapia, is considered the keystone farmed species and constitutes almost 80% of total tilapia aquaculture production in the world [1]. Tilapia can survive in unfavorable environments and is an excellent potential bioindicator for aquatic toxicity research [2, 3] and a model fish for studying toxicity in aquatic habitats [4, 5]. Unfortunately, aquaculture is exposed to pollutants that limit fish production and negatively impact fish growth and physiological homeostasis. Almost all industrial toxic effluents eventually end up in aquatic ecosystems and the frequent discharges of these pollutants have negative impacts on aquatic ecosystems [6, 7]. Moreover, along the coastal areas of the Egyptian Nile River increasing phenolic compounds residues and other petroleum components is considered a limiting factor for the survivability of fish species in different natural water which leads to elevating mortalities [8].

Plastic is one of the largest pressing environmental pollutants in the world and reaching plastic residue in marine water transforms the problem of plastic pollution from a local problem to a global one [9, 10]. As it’s known, plastic is a multiuse product that has chemical stability and is considered not expensive [9]. For all the mentioned plastic advantages, the production of plastic synthetic polymers increases without information about waste elimination strategies, especially with increasing the plastic demand during 2021 in industrial activities associated with the COVID-19 crisis [11]. Looking into the world’s future, if the growth rate of plastic polymers keeps the same, many predictions expected that by 2050 plastic amount may be 12,000 million tons pollute the environment [9].

Egypt is considered the largest consumer of plastic material in Africa, in 2017 is about 2.1 million tons [12]. Both microplastics (MPs) in the size range of 1–5000 μm and nano plastics in the size less than 1 μm are produced from the breakdown of the plastic particles. The small size of these particles makes their elimination from the ecosystem very difficult [13, 14]. Polystyrene nanoplastic (PS-NPs) is the mainly detected polymer of all NPs polymers in the environment [15]. The toxicity of PS-NPs in aquatic biota is due to their large surface area and small size which allow them to easily diffuse in water and penetrate into the tissues of organisms [16, 17]. Recently, many studies have demonstrated that fish are affected by PS-NP exposure, as it react with living hosts passing through biological membranes and then accumulated in their organs resulting in adversely influenced cell functions and causing various hazards such as oxidative damage, liver damage, developmental toxicity, and other neurobehavioral effects [18–21] and started pathological mechanisms by producing ROS and causing inflammation [22, 23].

Oil spills are major hazards to aquatic life and affect aquatic organisms by increasing their mortality and causing severe damage and health problems through bioconcentration [24]. Oil residues and other petroleum wastes enter water systems through shipments, oil refineries, oil rigs, or as a result of oil spill accidents from fishing ships and boats [24]. Crude oil is toxic and has a strong tendency to accumulate in the bodies of aquatic hosts [25]. Crude oil spills that occur underwater receive more attention than oil spills on the ground. This may be due to the rapid diffusion of floating oils by waves [26]. Changes in morphological, behavioral, biochemical, metabolic, and enzymatic parameters as well as oxidative stress were recorded in fish after exposure to crude oil or bioaccumulation of alkylphenols and PAHs in the bodies of aquatic organisms [27]. Laboratory experiments concerning the histopathological changes accompanied to crude oils exposure in fish [28, 29]. EO is used to lubricate various engines and enters the aquatic environment after rainfall [30].

Unfortunately, there is currently no information available that documents the harmful effects of combined PS-NPs and EO exposure in fish. Additionally, investigations conducted during exposure are necessary to provide a better understanding of the toxicity level of these substances. Thus, the current study sought to evaluate the toxicity of PS-NPs and EO as well as their synergistic effects on Nile tilapia, following a 15-day exposure period. Evaluations were conducted on hematobiochemical parameters, proinflammatory cytokines, oxidative stress indicators, and histological changes in vital organs.

## Materials and methods

### Polystyrene nanoplastics and engine oil

Polystyrene nanoplastics (PS-NPs) particles used in the present work with diameters (50.1 nm ± 13.4 SD) was purchased from Toxemerge Pty Ltd. (Melbourne, Australia). The mean diameter and size distribution that form polystyrene particles were determined by using Transmission Electron Microscopy (TEM) in a previous study [31]. The stock solution of PS-NPs used in the experiment with concentration of 5 mg/L was prepared directly before the experiment in deionized water [31]. Shell Helix Car Engine Oil from Shell, Egypt was used in this study.

The present work was conducted on 120 healthy Nile tilapia with a weight of about 98 ± 0.14 g and a length of about 12 ± 0.22 cm. These fish were collected from the aquaponic of Assiut university and were taken quickly to the fish biology lab at South Valley University. Fish were kept for acclimation in fiberglass tanks (160 L) for two weeks, these aerated freshwaters were de-chlorinated at 22.5 °C, pH = 7.4, dissolved oxygen = 6.9 mg/L and a photoperiod of 12 h light/dark. At the beginning of fish acclimation, fish undergo fasting for two days, then were fed one time daily (5% of the fish biomass) with a commercial fish diet purchased from Al-Nour company, Cairo. Experimental fish (*n =* 120) were distributed into 4 groups including 4 aquariums in 3 replicates per group (10 fish per aquarium). Group (i) contained fish in water free from any toxicant (control). Group (ii) contained fish that was exposed to 5 mg/L PS-NPs. Group (iii) contained fish that was exposed to 1% of EO [27]. Group (iv) contained fish that was exposed to (5 mg/L of PS-NPs + 1% of EO). During the 15 days of the experiment, water was replaced every day with about 50% of the tank’s water. To decrease the water ammonia, feces, and wastes were eliminated by siphoning off every day.

### Blood sample collection

Following a 15-day exposure period, six fish per group were netted out and used for blood sampling after anesthesia using ice [32]. A suitable quantity of blood was carefully drawn from the caudal vein of fish by using a 1 mL syringe. Then, a part of the blood was kept in special tubes containing an anticoagulant substance for hematological parameters. The other part of the blood specimen was kept in vacutainer tubes free from anticoagulant and centrifuged at 3000 rpm for 15 min to separate serum for analysis of proinflammatory cytokines, antioxidant enzymes and biochemical parameters.

### Hematological parameters

By using an automated analyzer (Mindray BC-2800) red blood cells (RBCs) count, hematocrit level (Ht), hemoglobin level (Hb), white blood cells (WBCs) count, differential leucocytic count and thrombocytes were estimated. In addition to erythrocyte indices, including mean corpuscular volume (MCV), mean corpuscular hemoglobin (MCH), and mean corpuscular hemoglobin concentration (MCHC) were estimated according to **Fazio et al.** [33].

### Proinflammatory cytokines

Interleukin-1β and IL-6 levels in the serum were measured by commercial ELISA kits with high sensitivity (Human Ultrasensitive, BioSource International Inc.).

### Oxidative stress biomarkers (antioxidant and lipid peroxidation parameters)

The activity of total antioxidant capacity (TAC) in serum samples was measured by method according to **Nishi­kimi et al.** [34]. Serum reduced glutathione (GSH) was also measured using commercial test kit (Biodiagnostic, Egypt, Catalog number: GR 2511), and as per manufacturer’s instructions. Level of Malondialdehyde (MDA) was determined by a thiobarbituric acid reaction [35].

### Biochemical parameters

The alanine aminotransferase (ALT), aspartate aminotransferase (AST) activities, creatinine, and uric acid levels were measured by using test kits (Biodiagnostic, Giza, Egypt).

### Histopathological examination

Fresh biopsies were dissected from the gills, liver, anterior and posterior kidney, and spleen and immediately fixed in 10% neutral buffered formalin, further dehydrated in ascending grades of alcohol, and clearance in xylene [36]. Paraffin sections of 5 μm thickness were stained by Haematoxylin and eosin (H&E) for microscopic imaging.

### Data analysis

For performing statistical analysis, SPSS software version 21.0 was used. To compare between the control group and the other exposed groups, one-way analysis of variance (ANOVA) was applied. Next, fisher’s least significant difference (LSD) post-hoc was used. The current results were recorded as mean ± SE with significance set at *p* < 0.05.

## Results

### Clinical signs and mortality

In the present study, PS-NPs exposure had no effect on fish health, survival rate was 100% and food intake and fish behavior were identical in both control and exposed groups. While fish exposed to either EO or the combination of PS-NPs + EO showed changed behavior, lower activity, and they showed erratic swimming, lack of normal reflex and then die. Moreover, small oil droplets were noticed on the surface of aquarium water.

The first record of mortality was obtained after 24 h of exposure of the experiment in both EO and PS-NPs + EO groups after struggling or standing vertically upward and gasping for air. Accounts of abnormal swimming and mortality with maximum level of 30% were noticed in PS-NPs + EO group. Whereas EO exposed group recorded 16.7% mortality.

Surviving fish were static and, gathered mostly near the aquarium edges, exhibited a change in behavior, erratic swimming pattern, and loss of reflexes.

### Hematological parameters

Results of hematological parameters of all exposed groups showed in **(**Table 1**).***O. niloticus* exposed to PS-NPs and / or EO has a significant decline in RBCs count, Hb, and Ht levels (p ˂ 0.05) when compared with the control group. Moreover, the interaction of both PS-NPs and EO exhibited a significant reduction in both MCH and MCHC in comparison to all other groups. Results here revealed that the combination group showed the maximum decrease in RBCs, Hb, MCH and MCHC than all other experimental groups.

Thrombocytes recorded a significant decline in PS-NPs and PS-NPs + EO intoxicated groups. In the same manner, in comparison to the control WBCs, and lymphocyte levels recorded a significant decline in PS-NPs intoxicated group, whereas PS-NPs + EO intoxicated group revealed a significant increase. Neutrophil count declined significantly in EO and PS-NPs + EO intoxicated groups than the control group.

### Proinflammatory cytokines

**Fig. 1:**
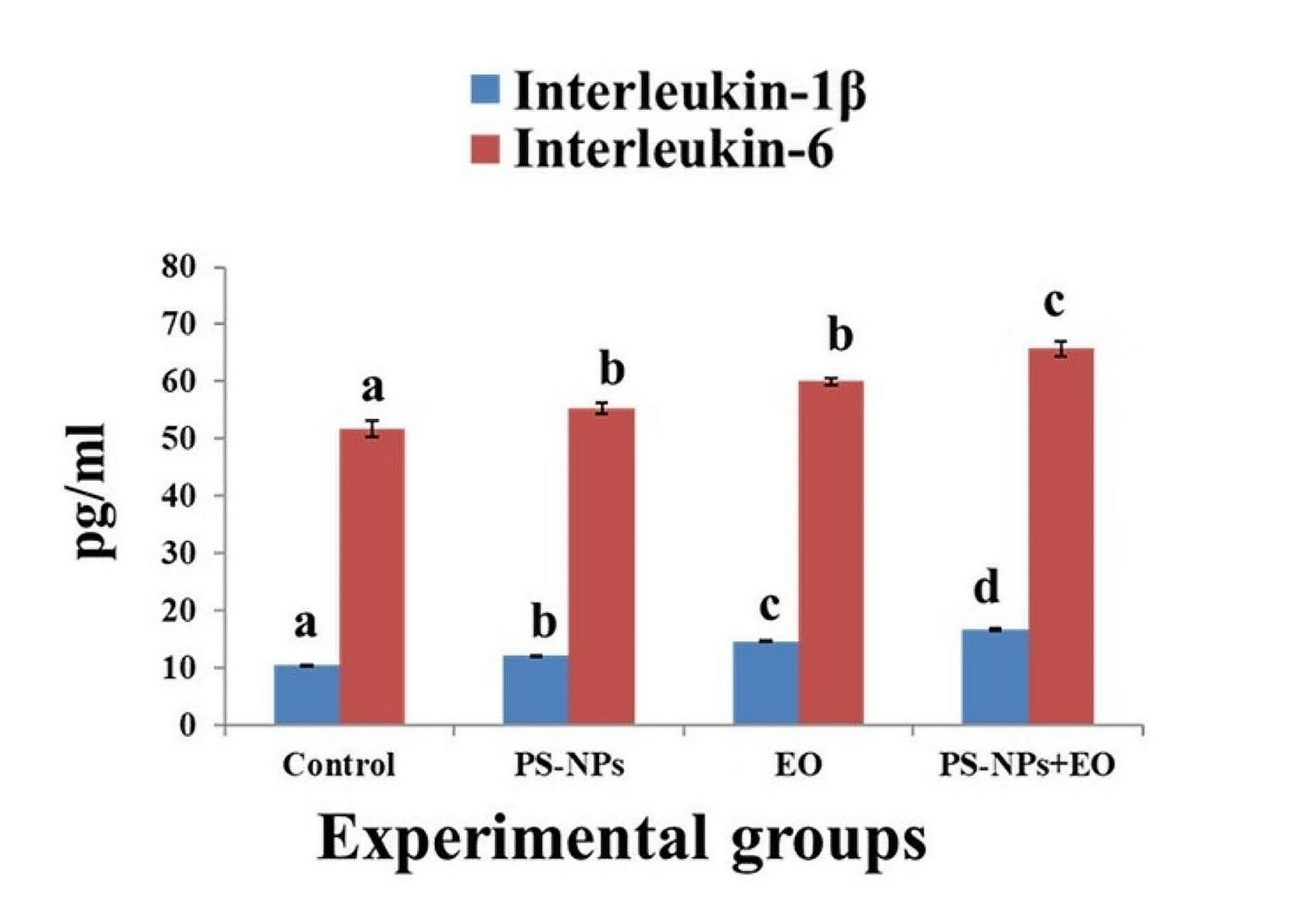
Proinflammatory cytokines results of Nile tilapia exposed to PS-NPs and / or EO for 15 days. Data are presented as the mean ± standard error (*n =* 6). Bars on the graph with different lowercase letters indicate significant difference between groups within each parameter (*P* < 0.05)

Serum 1 L-1β and 1 L-6 revealed significant elevation in all fish exposed groups in respect to control **(**Fig. 1**)**. The combination group showed the maximum elevation in the two measured cytokines than all other groups.

### Oxidative stress biomarkers

**Fig. 2:**
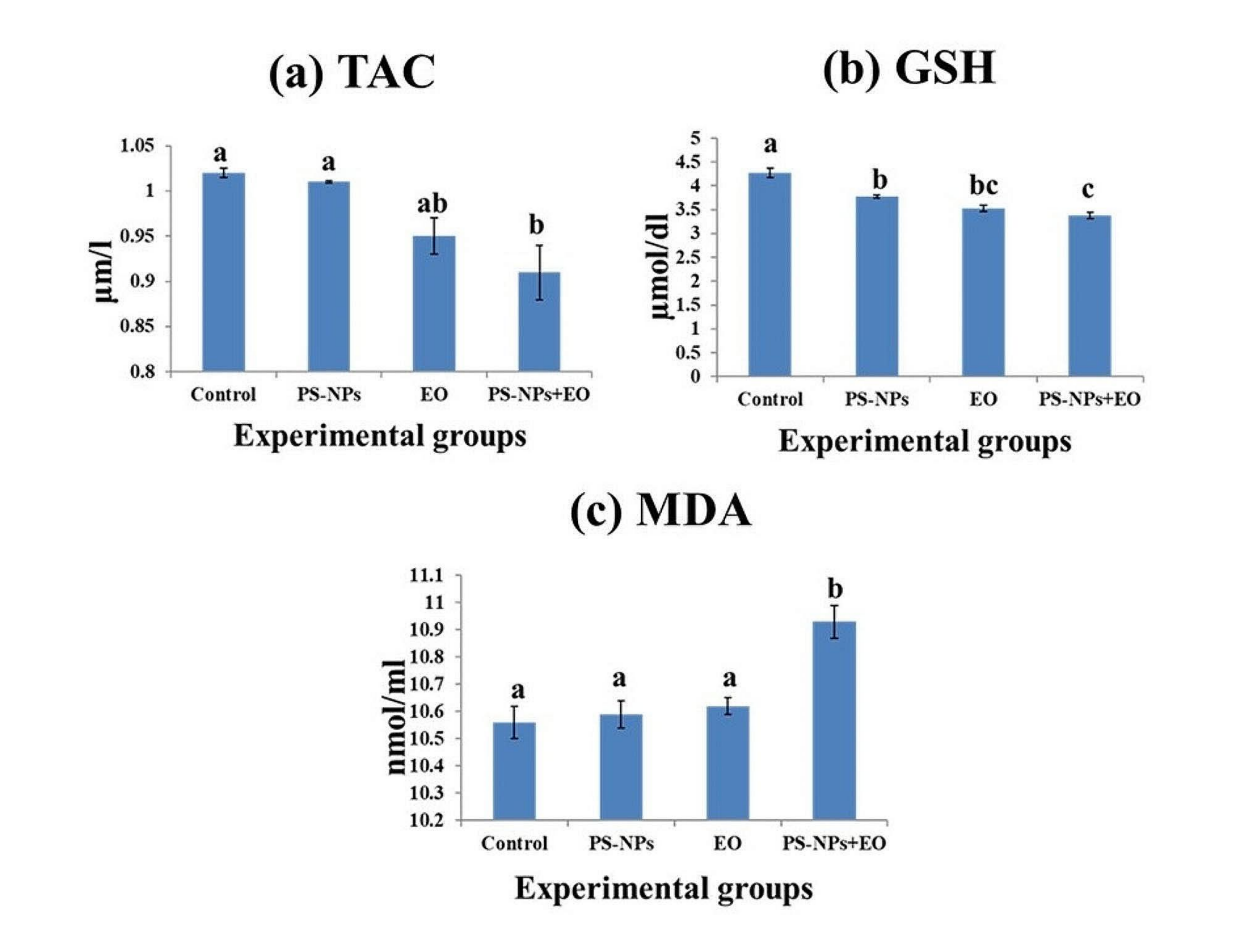
Oxidative stress parameters of Nile tilapia exposed to PS-NPs and / or EO for 15 days. (**a**) Total antioxidant capacity (TAC), (**b**) Reduced glutathione (GSH), and (**c**) Malondialdehyde (MDA). Data are presented as mean ± standard error (*n =* 6). Bars on the graph with different lowercase letters indicate significant difference between groups (*P* < 0.05)

The activity of TAC displayed a remarkable decrease (*p* < 0.05) in PS-NPs + EO combined group and MDA showed a remarkable increase in the same group, while the GSH activity recorded a significant decline in all experimental groups **(**Fig. 2**)**.

### Biochemical parameters

Results of liver and kidney biochemical responses of a 15-day exposure of *O. niloticus* to PS-NPs and / or EO are presented in **(**Table 2**)**. Only the PS-NPs + EO intoxicated group significantly increased in serum AST activity when compared to the control, while ALT revealed non-significant variation in all exposed groups.

In comparison to the control, uric acid and creatinine showed significant high levels in the intoxicated groups (*p* < 0.0001) except in PS-NPs intoxicated group for creatinine level, with uric acid showed the maximum elevation in the combination group than all other groups.

### Histopathological results

The histopathological findings proved that exposure to PS-NPs and / or EO produced significant destructive damage in various target organs varying according to severity and the combination group had the more sever histopathological alterations. The pathomorphological damage included degeneration, necrosis, congestion of the blood vessels and inflammatory conditions as presented in **(**Table 3**)**.

### Histopathological alterations of the gills

**Fig. 3:**
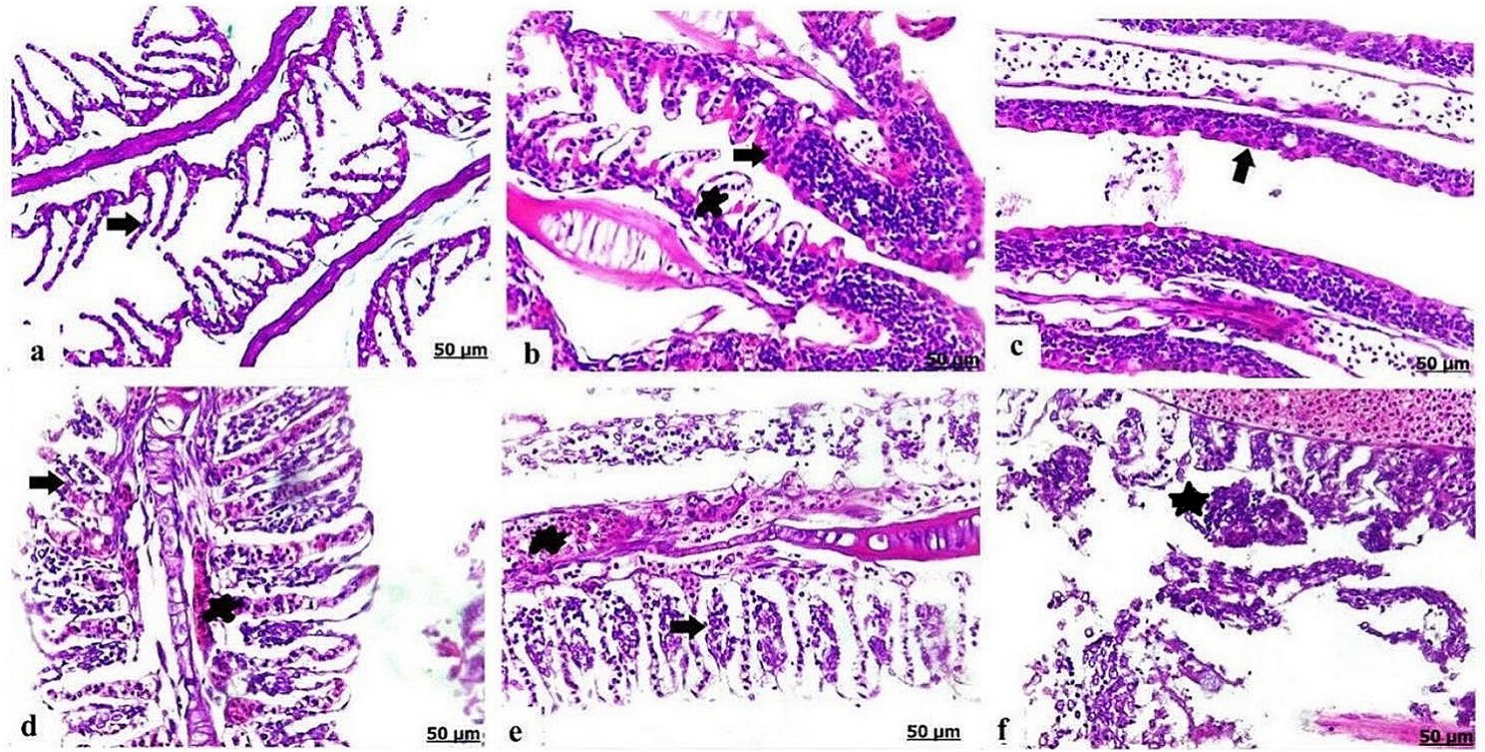
Light photomicrograph of gills of control and exposed Nile tilapia sectioned with H&E stain: Control showing normal histological architecture comprising normally arranged gill lamellae (arrow) **(a)**. The gills of PS-NPs exposed group showing hyperplasia of epithelial lining lamellae led to fusion of secondary lamellae (arrow), besides vacuolization of secondary lamellae (star) **(b)**, sloughing and loss of secondary lamellae (arrow) **(c)**. The gills of EO exposed group showing necrosis of gill lamellae (arrow), besides congestion of blood vessels (star) **(d)**. Gills of PS-NPs + EO exposed group showing necrosis and vacuolation of gill lamellae (arrow), moreover congestion and dilation of central venous sinus (star) **(e)**, necrosis with sloughing and desquamation of secondary lamellae (arrow) **(f)**

Control *O. niloticus* exhibited normal histology of gills lamella and gill filaments lined with intact epithelium **(**Fig. 3a**)**. While fish exposed to PS-NPs demonstrated thickening and hyperplasia of epithelial lining of lamellae resulted in the incomplete fusion of the secondary lamellae, besides expanded central venous sinus **(**Fig. 3b**)**, additionally necrosis with complete sloughing and loss of secondary lamellae were detected **(**Fig. 3c**)**. Gills of EO exposed group displayed necrosis of the gill lamellae and congestion of the blood vessels **(**Fig. 3d**)**. The combined PS-NPs + EO exposed fish showed severe necrosis and vacuolation of the epithelial lining lamellae, besides congestion and dilation of the central venous sinus **(**Fig. 3e**)**, moreover necrosis resulted in sloughing and desquamation of secondary lamellae **(**Fig. 3f**)**.

### Histopathological alterations of the liver

**Fig. 4:**
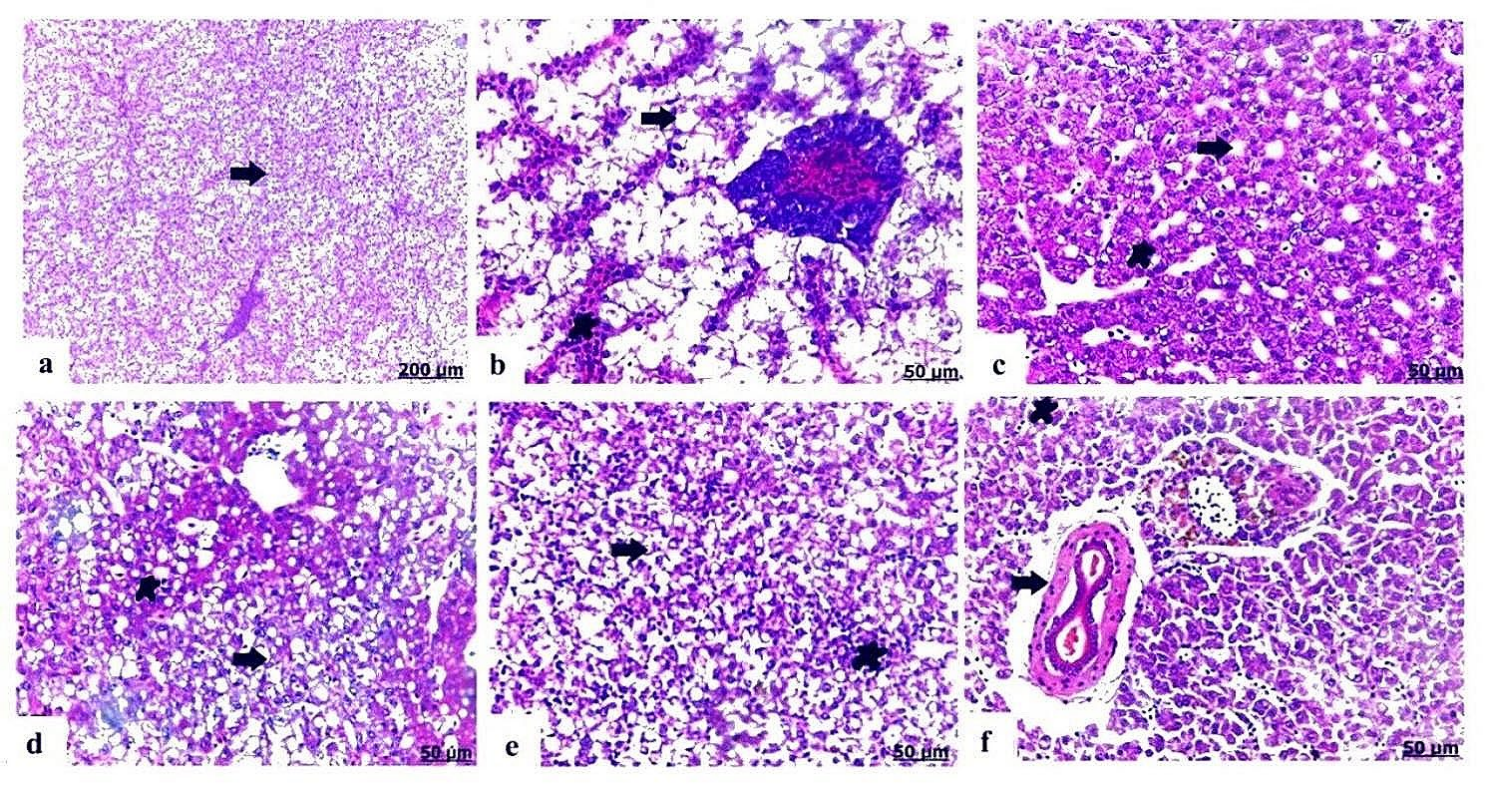
Light photomicrograph of liver of control and exposed Nile tilapia sectioned with H&E stain: Control showing normal histological structure of the hepatic parenchyma (arrow) **(a)**. Liver of PS-NPs exposed group showing vacuolation of the hepatocytes (arrow), and blood vessels congestion (star), **(b)**, and hepatic necrosis (arrow) with lymphocytes infiltration (star) **(c)**. Liver of EO group showed clear vacuoles in hepatocytes (arrow), besides minimal interstitial leucocytes infiltration (star) **(d)**. Liver of PS-NPs + EO exposed group showing noticeable degree of hepatic necrosis (arrow), besides focal accumulation of mononuclear cells (star) **(e)**, thickening and congestion of blood vessels (arrow) and lymphocyte infiltration (star) **(f)**

Liver of the control *O. niloticus* showed normal histological criteria of the hepatic parenchyma comprising of healthy hepatocytes and blood vessels **(**Fig. 4a**)**. Fish exposed to PS-NPs detected fatty infiltration of hepatocytes and hepatic necrosis with mononuclear cells infiltration mainly lymphocytes **(**Fig. 4b); furthermore, there was vacuolar degeneration of the hepatocytes characterized by cytoplasmic vacuolation, besides congestion of the blood vessels **(**Fig. 4c**)**. As well as EO exposed group manifested clear vacuoles of fat deposited within hepatocytes, and necrosis of the hepatocytes distinguished with lymphocytes infiltration **(**Fig. 4d**)**. Regarding to liver of the combined PS-NP + EO exposed group, it pronounced loss of hepatic arrangement and organization, hepatic necrosis characterized with focal aggregation of mononuclear cells mainly lymphocytes **(**Fig. 4e**)**, moreover congested and dilated blood vessels **(**Fig. 4f**)**.

### Histopathological alterations of the spleen

**Fig. 5:**
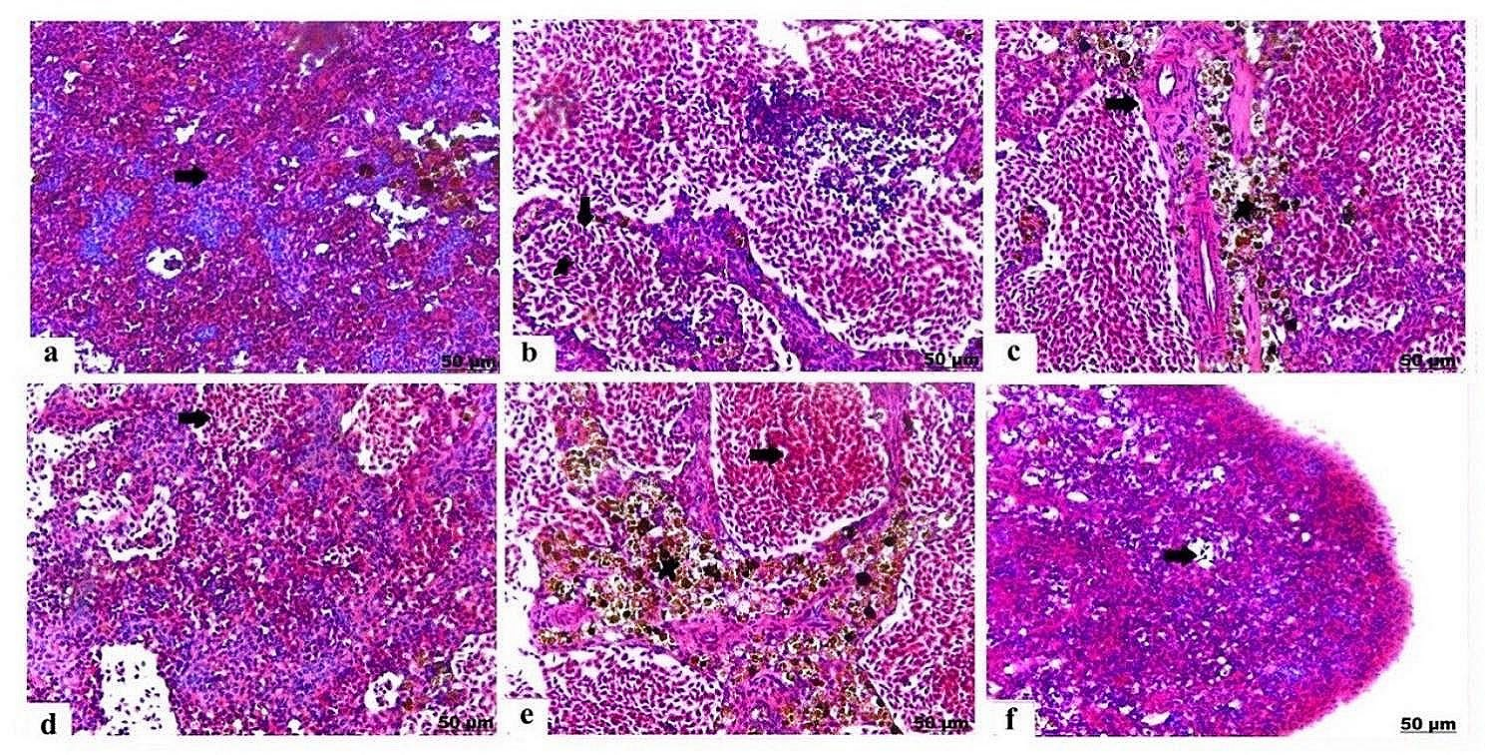
Light photomicrograph of spleen of control and exposed Nile tilapia sectioned with H&E stain: Control showing normal histology of the splenic red and white pulps (arrow) **(a)**. The spleen of PS-NPs exposed group showing severe hemorrhage of the red pulps (arrow) with erythrocytes infiltration (star) **(b)**, besides remarkable thickening of blood vessels wall (arrow) and melanomacrophages accumulation (star) **(c)**. The spleen of EO exposed group showing pronounced hemorrhage with erythrocytes infiltration (arrow) **(d)**. The spleen of PS-NPs + EO group showing extensive hemorrhage of the red pulps (arrow), moreover melanomacrophages infiltration (star) **(e)**, besides lymphoid depletion and vacuolation (arrow) **(f)**

Histological section of control spleen stained with H&E included normal parenchyma of the splenic pulps **(**Fig. 5a**)**. Unlike, spleen of PS-NPs exposed group exhibited noticeable hemorrhage of the red pulps characterized by erythrocytes infiltration **(**Fig. 5b**)**, besides this distinct thickening in blood vessels wall was detected, and melanomacrophages infiltration **(**Fig. 5c**)**. EO exposed group manifested hemorrhage in the red pulps **(**Fig. 5d**)**. In case of PS-NPs + EO group, spleen showed intense hemorrhage in the red pulps, and prominent accumulation of melanomacrophages **(**Fig. 5e**)**; moreover, there was depletion and decrease of the lymphoid population in the white pulps **(**Fig. 5f**)** and thickening of the splenic capsule.

### Histopathological alterations of the kidney

**Fig. 6:**
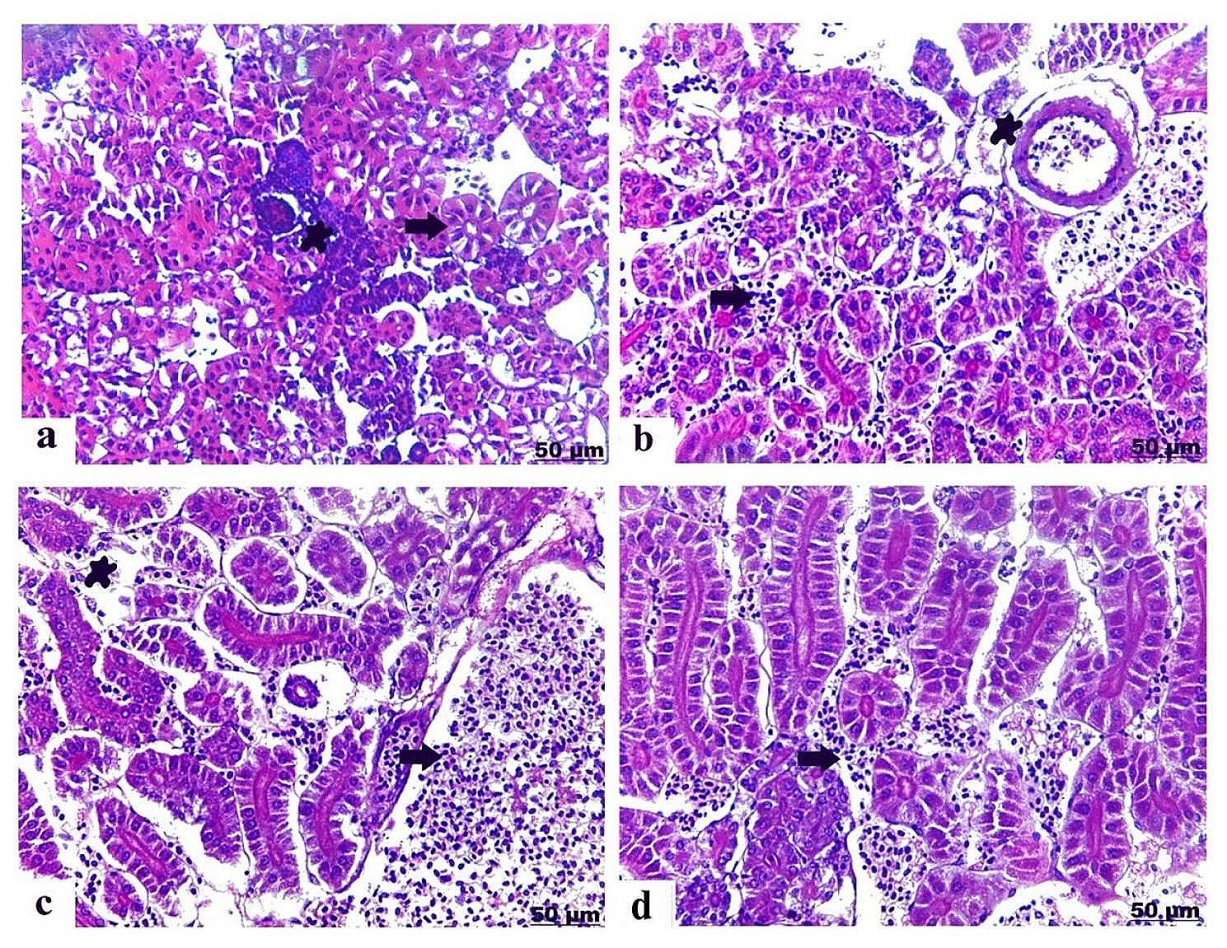
Light photomicrograph of head kidney of control and exposed Nile tilapia sectioned with H&E stain: Control head kidney showing normal renal parenchyma with intact tubules (arrow) and hematopoietic tissues (star) **(a)**. The head kidney of PS-NPs exposed group showing extensive thickening and congestion of the blood vessels (star), besides marked lymphocytes infiltration (arrow) **(b)**. The head kidney of EO exposed group showing distinct congested blood vessels (arrow) and necrosis with disappearance of the nephritic tubules (star) **(c)**. Head kidney of PS-NPs + EO exposed group showing hemorrhagic inflammation of hematopoietic tissues characterized by mixed infiltration with lymphocytes and erythrocytes (arrow) **(d)**

Section of control head kidney revealed normal histology of the hematopoietic tissues and renal corpuscles **(**Fig. 6a**)**. Contrariwise, PS-NPs induced group showed severe congestion and thickened and dilated blood vessels, besides hemorrhage with RBCs infiltration and necrosis with depletion of the hematopoietic tissues **(**Fig. 6b**)**. Likewise, head kidney of the EO exposed group detected congested blood vessels, besides necrotic changes in the tubules **(**Fig. 6c**)**, as well degenerative changes with renal tubular vacuolation were detected. In the head kidney that received PS-NPs + EO; there was interstitial hemorrhage characterized by red blood cells infiltration **(**Fig. 6d**)**.

**Fig. 7:**
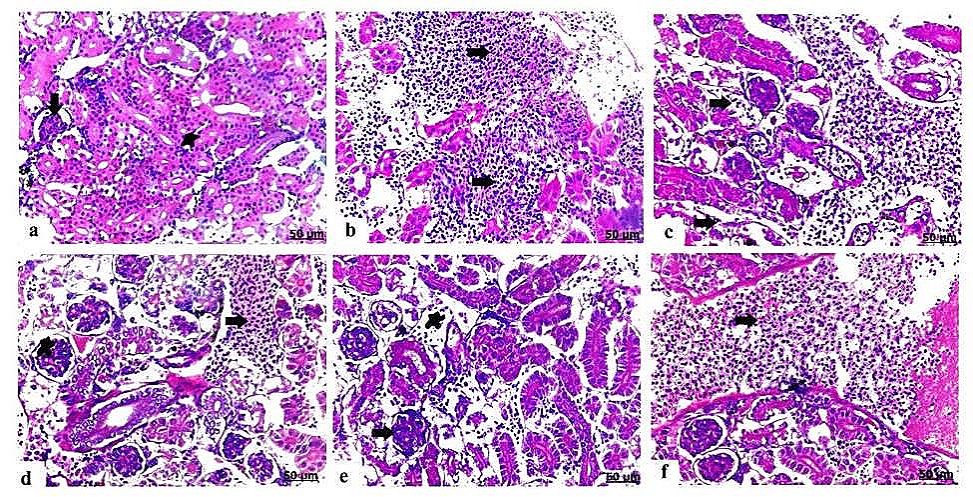
Light photomicrograph of trunk (posterior) kidney of control and exposed Nile tilapia sectioned with H&E stain: Control showing normal histology of the renal corpuscles composed of normal glomeruli (arrow) and renal tubules (star) **(a)**. The trunk kidney of PS-NPs exposed group showing severe necrosis with hemorrhagic inflammation of the renal tubules (arrows) **(b)**, necrosis of the renal tubules (arrows) **(c)**. The trunk kidney of EO exposed group showing congestion of the renal blood vessels (arrow), and glomeruli (star) **(d)**. Trunk kidney of PS-NPs + EO exposed group showing hypercelllularity and congestion of the glomeruli (arrow), in addition lymphocytes and erythrocytes infiltration (star) **(e)**, besides blood vessels severe congestion and engorgement (arrow) **(f)**

Meanwhile, control trunk (posterior) kidney had normal histological structure of the renal corpuscles included normal glomeruli and renal tubules **(**Fig. 7a**)**. Contrary to the kidney of PS-NPs exposed group manifested prominent necrosis with hemorrhagic inflammation of the renal tubules **(**Fig. 7b**)**, in addition to intensive congested and dilated renal blood vessels **(**Fig. 7c**)**. Likewise, trunk kidney of EO exposed group displayed renal dilatation and congestion of the tubular and glomerular blood vessels **(**Fig. 7d**)**. The possible effect of EO in trunk kidney received PS-NPs induced glomerular hypercellularity and blood vessels congestion **(**Fig. 7e **and f)**.

**Table 1:** Hematological parameters of Nile tilapia exposed to PS-NPs and / or EO for 15 days

Parameters	Control	PS-NPs	EO	PS-NPs + EO
RBCs (Million/mm^3^)	1.98 ± 0.005^a^	1.84 ± 0.02^b^	1.8 ± 0.02^b^	1.73 ± 0.006^c^
Hb (g/dl)	9.42 ± 0.02^a^	8.93 ± 0.11^b^	8.72 ± 0.06^b^	7.6 ± 0.07^c^
Ht (%)	25.25 ± 0.48^a^	24 ± 0.47^b^	23.62 ± 0.24^b^	23.14 ± 0.24^b^
MCV (µm³)	127.68 ± 2.41^a^	130.59 ± 2.88^a^	131.48 ± 1.96^a^	133.96 ± 1.51^a^
MCH (Pg)	47.66 ± 0.21^a^	48.59 ± 0.55^a^	48.56 ± 0.62^a^	43.98 ± 0.49^b^
MCHC (%)	37.36 ± 0.67^a^	37.27 ± 1.05^a^	36.94 ± 0.41^a^	32.84 ± 0.36^b^
Thrombocytes (Thousands/mm^3^)	314.5 ± 1.32^a^	302.25 ± 1.1^bc^	310.5 ± 0.96^ab^	295.75 ± 4.5^c^
WBCs (Thousands/mm^3^)	841.75 ± 3.09^a^	816 ± 3.65^b^	839 ± 4.7^a^	862 ± 3.24^c^
Neutrophils (%)	6.75 ± 0.25^a^	5.75 ± 0.25^ab^	5 ± 0.4^b^	5 ± 0.4^b^
Lymphocytes (%)	88.5 ± 0.5^a^	86.25 ± 0.48^c^	89.25 ± 0.25^a^	90 ± 0.58^b^
Monocytes (%)	3.25 ± 0.25^a^	3 ± 0.4^a^	2.75 ± 0.25^a^	2.5 ± 0.29^a^

Data are presented as the mean ± standard error (*n =* 6). Row with different superscript letters indicated significant difference between groups (*P* < 0.05)

**Table 2:** Biochemical parameters of Nile tilapia exposed to PS-NPs and / or EO for 15 days

Parameters	Control	PS-NPs	EO	PS-NPs + EO
AST (U/ml)	55.45 ± 0.05^a^	55.35 ± 0.93^a^	55.92 ± 0.43^a^	57.82 ± 0.72s^b^
ALT (U/ml)	29.75 ± 0.48^a^	29.07 ± 0.37^a^	30.95 ± 0.63^a^	30.7 ± 0.7^a^
Uric acid (mg/dl)	11.82 ± 0.02^a^	12.75 ± 0.13^b^	13.6 ± 0.07^c^	14.22 ± 0.2^d^
Creatinine (mg/dl)	0.64 ± 0.008^a^	0.67 ± 0.02^a^	0.8 ± 0.0^b^	0.82 ± 0.02^b^

Data are presented as the mean ± standard error (*n =* 6). Row with different superscript letters indicated significant difference between groups (*P* < 0.05)

**Table 3:** Histopathological scoring of H&E-stained sections categorized according to lesions degree of the gills, liver, spleen, and kidney of Nile tilapia exposed to PS-NPs and / or EO for 15 days

Groups	Control	PS-NPs	EO	PS-NPs + EO	
**Gills lesions**					
Necrosis and vacuolation of the gill lamellae	-	+++	+++	+++	
Sloughing and desquamation of lamellae	-	++	+++	+++	
Shortening and curling of secondary lamellae	-	++	++	++	
Fusion of secondary lamellae	-	++	++	++	
Inflammatory cells infiltration	+	++	++	++	
Expansion and congestion of central venous sinus	-	++	++	+++	
Congestion of the lamellar blood vessels	-	+++	++	++	
**Liver lesions**					
Necrosis of the hepatocytes	-	++	+++	++	
Hepatic cytoplasmic vacuolation	+	++	+++	++	
Fatty infiltration of the hepatocytes	-	++	++	+++	
Interstitial cells infiltration	-	++	++	++	
Congestion and dilatation of the blood vessels	+	+++	+++	+++	
**Spleen lesions**					
Hemorrhage of red pulps	-	+++	++	+++	
Lymphoid depletion of the white pulps	+	++	++	+++	
Melanomacrophages infiltration	-	+++	++	+++	
Protrusion of splenic trabeculae	-	++	++	++	
Thickening of splenic capsules	-	++	++	+++	
Congestion and dilatation of the blood vessels	-	++	++	++	
Thickening of the blood vessels wall	+	+++	+++	++	
**Kidney lesions**					
Renal necrosis	-	+++	++	++	
Degenerative changes of the renal tubules	+	++	++	+++	
Inflammatory cells infiltration	+	+++	+++	+++	
Hemorrhage of the hematopoietic tissues	+	+++	+++	+++	
Melanomacrophages infiltration	-	+++	++	+++	
Glomerular hypercellularity and congestion	+	++	+++	+++	
Glomerular shrinkage and atrophy	-	++	++	++	
Dilatation and congestion of the blood vessels	-	+++	++	+++	

Score: Absent, (-), mild (+), moderate (++), and severe (+++). PS-NPs: Polystyrene nanoplastic

## Discussion

Pollution from PS-NPs and EO are of major concern. The current study analyzed the negative impacts of PS-NPs and / or EO on several biomarkers of Nile tilapia to determine their synergistic toxicity. Earlier studies stated that nanomaterials can influence fish behavior through biochemical changes in the brain [18, 37, 38]. In our study, PS-NPs exposure did not affect fish performance, fish survival was 100%, and feed intake and fish behavior were comparable in control and exposed fish. Similar results recorded by Mattsson et al. [20], Brandts et al. [39]. This suggests that either the concentration of PS-NPs or the exposure time is important. It took several weeks before significant behavioral changes were observed, and our study lasted for 15 days. This confirms that fish are affected when PS-NPs accumulate in fish tissues and reach the brain. Meanwhile, fish exposed to EO showed behavioral changes compared to the control and small oil droplets were noticed on the water surface, these tiny oils slow the absorption of oxygen by the water, resulting in decreased dissolved oxygen levels and death of the fish after struggling and gasping for air. Similar results recorded by Akin-Obasola [30], Ugwu et al. [40], Solomon et al. [41]. Accounts of abnormal swimming and mortality with maximum level were noticed in the combined PS-NPs + EO group which attributed to EO is absorbed through the gills and it transferred to the brain and other organs through the blood.

Blood parameters are useful in diagnosing various diseases, assessing the health condition of fish and impact of various pollutants [33]. Generally, stressors (e.g. toxic compounds) induce alterations in blood cells of exposed fish. Exposure of Nile tilapia to PS-NPs and / or EO causes a significant decrease in almost all the measured blood parameters and the combination group showed extreme significant levels than all other experimental groups. Toxic substances have destructive toxic effects that damage the delicate RBCs membranes, cause hemolysis, and cause a decrease in cells, Hb, and Ht due to tissue oxygen deprivation [42, 43] and reducing both lifespan of RBCs and heme synthesis [44]. Inhibition of both erythropoiesis and Hb biosynthesis were recorded in Nile tilapia [32] and African catfish [45] after exposure to MPs and in Nile tilapia exposed to EO [46].

The decline in the MCH and MCHC of the combined PS-NPs + EO group is an indication of anemia [47] results from variations in RBCs shape, size and concentration of Hb [48]. Severe anemia recorded in the combined group confirms the suppression of erythropoiesis and cytotoxicity caused by PS-NPs and EO. Similar findings were recorded in *C. gariepinus* feed 45 days on polyvinyl chloride microparticles (PVC-MP) [45]. The level of thrombocytes decreased in fish intoxicated groups, PS-NPs, and PS-NPs + EO compared to the control. It has been known that toxic components impaired the thrombocytes and reducing their values [49].

The role of WBCs is to compact infection and initiate an immune response. Results here revealed fluctuation in WBCs count, it decreases after PS-NPs exposure and increases after PS-NPs + EO exposure. The decline in WBCs is considered a consequence of the PS-NPs toxicity on lymphoid tissues of intoxicated fish [50]. The side effect of crude oil is to activate the humoral immune response of the fish body which stimulates WBCs production to engulf the toxic substances [51] suggests that the Nile tilapia is exposed to higher risk of stress results from the combined effect of PS-NPs + EO exposure. The changes in various types of WBCs can serve as a sign for immunological changes in fish that are exposed to toxic pollutants [52]. In comparison to control, combined PS-NPs + EO had a decreased level of neutrophils and increased level of lymphocytes which oppose the results of Davis et al. [53]. It has been noted that all WBCs were calculated as a percentage of the total leucocytes (neutrophils, lymphocytes, monocytes, eosinophils, and basophils) which makes up 100%, as a result either decrease or increase in different leucocytes was observed in fish. The increase of the lymphocytes in PS-NPs + EO group may be attributed to the decrease of other leucocytic cells.

Since *O. niloticus* homeostasis is disrupted by exposure to PS-NPs and EO, this study anticipates that exposure to these substances would result in tissue damage, which is marked by an increase in lymphocytes, the influx of pro-inflammatory cytokines, and reactive oxygen species (ROS) [54]. When cellular homeostasis is disrupted by these toxicants, interleukins immediately trigger an immune response to this emerging stressor. This was confirmed here as fish exposed to either PS-NPs or EO and their combination suffered an increased IL-1β and IL-6 levels where the combination group recorded the maximum significant elevation in comparison to all other toxicity groups, which in case of inflammation and cellular damage are typical immune responses [55]. Similar inflammatory indications found in earlier studies [56] confirmed by an increased expression level of *IL-1β* gene in snakehead fish after exposure to nano-microplastics and cadmium and in *C. gariepinus* exposed to microplastics and lead [57]. Uncontrolled production of pro-inflammatory cytokines leads to serious systemic inflammatory response with pathological consequences [58] which was confirmed in the current histological alterations of vital organs.

Total antioxidant capacity is a reliable representation reflecting the overall condition of the antioxidant system [59]. In this study, the activity of TAC recorded a significant decrease after combined PS-NPs + EO exposure. This signifies that the antioxidant system is disrupted and cannot suppress the generated oxidative damage and confirms the synergy between toxicants used. Oxidative stress resulted when the ROS produced exceeds the protection supplied by the antioxidant enzymes such as superoxide dismutase, catalase, glutathione S-transferase, and GSH [60]. Meanwhile, in all treatment groups the GSH activity decreased compared to the control group, indicating disruption of redox homeostasis. GSH serves as a fundamental line of defense against the destructive effects of ROS and protects cells from oxidative damage [61]. This decrease in GSH recorded here may be due to increased utilization due to enhanced ROS generation [62]. Nanoplastics that cause imbalance in antioxidant enzymes have been observed in different fishes including juvenile *Macrobrachium nipponense* [63], Channel Catfish Larvae [64] and juvenile *Larimichthys crocea* [65] *C. gariepinus* [45]. In addition, oil derivatives result in significant reduction in the levels of glutathione, glutathione peroxidase and superoxide dismutase [66] due to oxidative stress condition that accompanied this pollutant. Whereas the activity of MDA showed a significant elevation only in the combined PS-NPs + EO group compared to all other groups. This increase in the MDA concentration observed here is an indicator of lipid peroxidation and indicates damage to the hepatocyte membrane. This is consistent with the opinion of Liu et al. [67] documented an increase in MDA due to phenanthrene exposure. Lipid peroxidation in cell membranes tends to damage polyunsaturated fatty acids and reduce membrane fluidity which is vital for cells function [61].

In consequence with previous findings, the present study documented that Nile tilapia exposed to combined PS-NPs + EO for 15 days suffered biochemical alterations, which confirmed their direct toxic effects. The activity of serum AST in fish increased in the PS-NPs + EO intoxicated group with respect to all other groups. The enzyme AST is primarily present in the cytoplasm of hepatic cells and can enter to bloodstream in cases of liver damage [68] or liver necrosis [69]. Liver damage from the accumulation of ROS or hepatic lipid peroxidation leading to an increase in hepatocyte permeability [70]. Under the influence of NPs, comparable results were seen in marine fish *Larimichthys crocea* [71], in juvenile common carp [72], and in *C. gariepinus* [31]. Additionally, EO and anthracene toxicity increase AST level in *O. niloticus* and *C. carpio* [46, 73]. In a similar trend, serum uric acid and creatinine were elevated in PS-NPs + EO exposed group and uric acid recorded the maximum significant elevation than all other groups. The presence of various pollutants in water bodies increases the formation of ROS, leading to damage to biological processes and systems [46, 74] and impaired the renal function [75]. Many previous studies have found similar results following MPs toxicity in *P. microps* [76], *O. niloticus* [32], and *C. carpio* [77]. Also, such elevation was recorded by Mohamed et al. [78] in Nile tilapia exposed to EO.

The present study showed a low level of RBCs, and Hb caused by the toxicants used, causing low oxygen level. Oxygen is critical to the fish for respiration through the gills, low levels of oxygen disturbing the circulation and causing respiratory affections. This respiratory failure may alter the fish tissue normal histology and function which was confirmed in the present histopathological examination of vital organs. It was recorded that histological alterations were attributed to hypoxia within tissues of fish [79]. In this study, higher levels of IL-1β as part of the defenses stimulate production of ROS [80]. Exposure to both MPs and NPs can increase the over-production of ROS, which disturbs the antioxidant defense and cause cellular damage [81, 82]. Excessive stress exposure led to increase the accumulation of ROS resulted in cell death, tissues injury, and inflammation [83, 84]. Under normal circumstances, antioxidant enzymes detoxify and remove ROS from the cells [85]. When there is an imbalance between the synthesis and removal of ROS, especially when the formation of ROS outpaces the antioxidant system, oxidative stress results [85]. Significant alterations in the antioxidant system’s functioning are a pathophysiological consequence of oxidative stress, and these modifications may cause harm to cells and tissues [86] which was confirmed in the present histopathological examination of vital organs.

Gills are extremely susceptible to all types of toxicants because of their wide surface area and constant and permanent uptake of water from the surrounding environment [87] during respiration. Because of this, it serves as a highly reliable bio-indicator of water pollution [88]. In PS-NPs exposed group, gills displayed intense histological alterations and structural cell damage at the sites of PS-NPs accumulation occurred. EO exposed fish gills suffered congestion of blood capillaries and aneurisms of the lamellae; similar findings recorded in *Tilapia zillii* and *Mugil cephalus* fishes exposed to burned motor oil for 45 days [89] and in *C. gariepinus* juveniles after exposure to different levels of premium motor spirit for 96 h [90]. The gills of the combination group showed sever damage with sloughing and desquamation of secondary lamellae attributed to the increased level of ROS and altered antioxidant enzyme [91] which was confirmed by low level of studied antioxidants. Also, alterations of the gills were owing to hypoxic condition and respiratory upsets [79] confirmed by the clinical signs appeared on exposed fish.

Hepatic damage is a typical sign in the liver of fish exposed to pollutants. The liver of the PS-NPs exposed group showed hepatocytes necrosis, inflammation, and vacuolar degeneration. Similar results were detected in *Carassius auratus* fish [92] after six weeks exposure to virgin MPs and in *Javanese medaka* fish exposed to PS-MPs for 3 weeks [93]. Exposure to NPs can promote excess produced ROS [94] and histological deterioration might occur [23]. Necrosis of the hepatocytes in the liver of EO exposed Nile tilapia are consistent with Agamy [95] in rabbit *Siganus canaliculatus* fish exposed to crude and dispersed oils for 21 days. In addition, the liver of EO exposed group revealed hepatic vacuolation as previously detected by Nwakanma and Hart [96] in *O. niloticus* exposed to EO for 21 days and by **Amadi et al.** [97] who detected hepatic degeneration, and inflammation in *C. gariepinus* exposed to refined petroleum oil. The significant rise in serum liver biomarker AST induced by PS-NPs + EO in the combination group was clarified by pathological findings in the hepatic tissue, where it revealed the extreme lesions than all other groups.

In the spleen, PS-NPs significantly interfere with antioxidant mechanisms causing excessive ROS production in cells [98]. Lesions in the spleen are distinguished by hemorrhages, thickening in the wall of blood vessels, and melanomacrophages infiltration. A decrease in lymphocytes with accumulation of MMCs were noticed in spleen of EO exposed fish as mentioned by Ali et al. [99]. Moreover, exposure to EO promoted depletion in lymphoid follicle of spleen, a variable number of melanomacrophages infiltrations and congestion of the blood sinusoids of spleen. Laboratory investigations discussed oil spills exposure leading to immunosuppression and lymphopenic status [100]. These findings suggest that EO results in cell and tissue disruption of immune organs that predispose fish to infectious diseases. The combined PS-NPs + EO group showed sever hemorrhage and lymphoid depletion which could interpret the migratory function of lymphocytes from lymphoid organs notably spleen and the absolute numbers of circulating lymphocytes [101].

In the same manner, Kidney histological alterations were characterized by necrosis of the epithelial lining of the renal tubules, and depletion of hematopoietic tissues, besides congestion in the blood vessels and the combined group showed the more sever degenerative changes in the renal tubules which suggest renal dysfunction [102]. Usman et al. [93] noted destructive damage and necrosis of the renal tubules and edema with congestion of the blood vessels in PS-MPs exposed *Javanese medaka*. The significant rise in serum kidney biomarkers, urea and creatinine induced by toxicant was clarified by pathological findings in the renal tissue. PS-NPs could instigate nephrotoxicity associated with higher levels of ROS [103].

## Conclusion

In conclusion, PS-NPs and / or EO exposure causes a negative impact on the health of Nile tilapia. The finding supports that the combination of PS-NPs and EO have synergistic toxic effect. Results confirm their toxic effect, through analyzing a set of hematobiochemical, proinflammatory cytokines, and oxidative stress parameters. Moreover, Histopathological alterations detected confirmed the toxicity and hazardous effects of these pollutants.

## Data Availability

All relevant raw data will be freely available from the authors.
